# Polymer models of chromatin organization

**DOI:** 10.3389/fgene.2013.00113

**Published:** 2013-06-20

**Authors:** Mariano Barbieri, Antonio Scialdone, Andrea Piccolo, Andrea M. Chiariello, Ciro di Lanno, Antonella Prisco, Ana Pombo, Mario Nicodemi

**Affiliations:** ^1^Dipartimento di Fisica, INFN Sezione di Napoli, CNR-SPIN, Universita' di Napoli Federico IINapoli, Italy; ^2^Computational and Systems Biology, John Innes Centre, Norwich Research ParkNorwich, UK; ^3^CNR Institute of Genetics and Biophysics “Buzzati Traverso”Naples, Italy; ^4^M. Delbrück Center for Molecular Medicine, Berlin Institute for Medical Systems BiologyBerlin-Buch, Germany

The exploration of the spatial organization of chromosomes in the cell nucleus has been greatly enhanced by genome-scale technologies such as Hi-C methods. Polymer models are helping to understand the new emerging complex scenarios and here we review some recent developments.

In the cell nucleus of eukaryotes, chromosomes have a complex spatial organization serving vital functional purposes, with structural disruptions being linked to disease (Fraser and Bickmore, 2007; Lanctot et al., 2007; Misteli, 2007; Pombo and Branco, 2007). The development of technologies such as Hi-C (Lieberman-Aiden et al., 2009) has opened the way to mapping chromatin interactions at a genomic scale. It is emerging that chromosomes tend to form 1Mb sized domains with increased levels of intra-interactions (known, e.g., as Topological Domains, TDs) (Dixon et al., 2012; Nora et al., 2012), but contacts extend across entire chromosomes (Branco and Pombo, 2006; Shopland et al., 2006; Fraser and Bickmore, 2007; Kalhor et al., 2011; Sexton et al., 2012), as highlighted by the average contact probability of two sites, *P*_*c*_(*s*), which is non-zero also for large genomic separations, *s*. In particular, in the 0.5–7 Mb range, *P*_*c*_(*s*) is found to decrease roughly as a power law with *s* (Fraser and Bickmore, 2007), *P*_*c*_(*s*) ~1/*s*^*α*^. Relevant inter-chromosomal contacts also exist (Branco and Pombo, 2006; Shopland et al., 2006; Lieberman-Aiden et al., 2009; Kalhor et al., 2011; Dixon et al., 2012; Nora et al., 2012; Sexton et al., 2012). Interestingly, while the map of genomic contacts has a stochastic component, a clear non-random organization is observed, which is cell-type and chromosome specific.

Discoveries as those listed above have raised fundamental questions on how such complex patterns self-organize, how functional, distal contacts can be reliably established, and how specific structures, like TDs, are assembled. Polymer physics has been employed to try to clarify some of those questions with models aiming at identifying the key physical elements involved. Here we do not attempt to cover the broad literature on the topic, which is summarized in previous reviews (Langowski, 2006; Marenduzzo et al., 2006; Emanuel et al., 2009; Tark-Dame et al., 2011), but we focus on a few recent developments triggered by the new experimental data.

The average contact probability, *P*_*c*_(*s*), was originally reported for a single human cell line to have an exponent α = 1.08 (Lieberman-Aiden et al., 2009), which is not found in usual *equilibrium* polymer systems [see (Langowski, 2006; Marenduzzo et al., 2006; Emanuel et al., 2009; Tark-Dame et al., 2011) and references therein]. That led to the idea that chromosomes are in a far-from-equilibrium state and, more precisely, in a specific *transient state* of ideal polymer chains, named the *Fractal Globule* (FG) state (Lieberman-Aiden et al., 2009), known to have an exponent α = 1. As the FG state is free of knots, it would allow a better control of chromosome conformations. However, if the effects of DNA cutting enzymes, such as topoisomerase, are considered in the model the FG state is not formed at all (Mirny, 2011).

More recent Hi-C data (Shopland et al., 2006; Kalhor et al., 2011; Dixon et al., 2012; Nora et al., 2012; Sexton et al., 2012) have clarified that the shape of *P*_*c*_(*s*), as much as its exponent, α, are system-dependent. We highlighted in a recent paper (Barbieri et al., 2012) that in human cell lines (Lieberman-Aiden et al., 2009; Kalhor et al., 2011; Dixon et al., 2012), for instance, α ranges roughly from 0.9 to 1.7, with different chromosomes in a given system having different exponents. The exponent α also changes across different organisms: in Drosophila (Sexton et al., 2012) α = 0.85 (with α = 0.7 in closed genomic regions). These experimental results have raised additional questions on how different conformations can be established and controlled according to the different circumstances.

To describe such a range of architectures, it was considered that chromosome 3D conformations are shaped by the interactions of chromatin with the nuclear envelope, with nuclear bodies and with DNA binding molecular factors. The latter case was explored within a schematic polymer model, the *Strings and Binders Switch* (SBS) model where chromatin is represented as a Self-Avoiding-Walk (SAW) chain having binding sites for diffusing molecules (Nicodemi et al., 2008; Nicodemi and Prisco, 2009; Barbieri et al., 2012). A similar scenario was also discussed in another polymer model, the *Dynamic Loop Model* (Bohn and Heermann, 2010), where the beads of the polymer have a probability to stick together when they randomly collide by diffusion.

The SBS model has revealed that the values of α reported in Hi-C experiments reflect genome-wide averages over heterogeneous, differently folded regions, organized in architectural classes, which can be well described by usual polymer physics (de Gennes, 1979; Doi and Edwards, 1984) as thermodynamic phases. A variety of off-equilibrium conformations also exists, which include the FG scenario. The SBS model (Barbieri et al., 2012) helped to reconcile in a unifying framework other experimental results such as single cell FISH data on the mean square spatial distance of pairs of loci, <*R*^2^>, and their moment ratio <*R*^4^>/<*R*^2^>^2^, in agreement with available data (Mateos-Langerak et al., 2009; Barbieri et al., 2012). It clarified how polymer architectural patterns can be established by randomly diffusing binding molecules, how different stable conformations can be produced, and how architectural changes can be reliably regulated by usual cell strategies, such as protein up-regulation or epigenetic chromatin modification, by exploiting fundamental thermodynamic mechanisms. Within the SBS model the formation of TDs domains, or the looping out of specific loci, can be rationalized by specialization of polymer binding sites and binding molecules (Barbieri et al., 2012). The SBS model has been criticized for the use of limited length polymer sizes (in the original version it was 512 bead long), but it was tested up to sizes of the same order of magnitude of those considered for the FG model without finding relevant differences. This is expected, in fact, for the scaling properties of polymer physics (de Gennes, 1979; Doi and Edwards, 1984).

In brief, the emerging picture is that chromatin is a complex, heterogeneous mixture of differently folded regions, self-organized across spatial scales by fundamental thermodynamics mechanisms. Models like those mentioned represent strong simplifications of the complexities arising in real nuclei. However, polymer scaling theory (de Gennes, 1979; Doi and Edwards, 1984) ensures that the general behavior of folding is independent of the system minute details and reflects universal properties, as those captured by the SBS model. Other effects, such as crowding and entanglement, should also be considered, as in the study of other complex fluids (Cataudella et al., 1994; Caglioti et al., 1998; Coniglio and Nicodemi, 2000; Nicodemi and Jensen, 2001; Tarzia et al., 2004). The SBS model has also been employed to describe specific chromosomal loci, such as the *Xist* locus (Nicodemi and Prisco, 2007; Scialdone et al., 2011a,b) or chromosome conformation at meiosis (Gerton and Hawley, 2005; Nicodemi et al., 2008). In summary, while many aspects of chromatin organization still remain obscure, simple polymer models are starting to clarify the fundamental physical mechanisms underlying its complex, stochastic, yet non-random patterns.

## References

[B1] Barbieri M. Chotalia M. Fraser J. Lavitas L.-M. Dostie J. Pombo A. (2012). Complexity of chromatin folding is captured by the strings and binders switch model. Proc. Natl. Acad. Sci. U.S.A. 109, 16173–16178 10.1073/pnas.1204799109 22988072 PMC3479593

[B2] Bohn M. Heermann D. W. (2010). Diffusion-driven looping provides a consistent framework for chromatin organization. PLoS ONE 5:e12218 10.1371/journal.pone.0012218 20811620 PMC2928267

[B3] Branco M. R. Pombo A. (2006). Intermingling of chromosome territories in interphase suggests role in translocations and transcription-dependent associations. PLoS Biol. 4:e138 10.1371/journal.pbio.0040138 16623600 PMC1440941

[B4] Cataudella V. Franzese G. Nicodemi M. Scala A. Coniglio A. (1994). Critical clusters and efficient dynamics for frustrated spin models. Phys. Rev. Lett. 72, 1541–1544 10.1103/PhysRevLett.72.1541 10055635

[B5] Coniglio A. Nicodemi M. (2000). The jamming transition of granular media. J. Phys. 12, 6601–6610 10.1088/0953-8984/12/29/331 16089733

[B6] Caglioti E. Coniglio A. Herrmann H. J. Loreto V. Nicodemi M. (1998). Segregation of granular mixtures in presence of compaction. Europhys. Lett. 43, 591–597 10.1209/epl/i1998-00402-x

[B7] de Gennes P. G. (1979). Scaling Concepts in Polymer Physics. Itaca, NY: Cornell University Press

[B8] Dixon J. R. Selvaraj S. Yue F. Kim A. Li Y. Shen Y. (2012). Topological domains in mammalian genomes identified by analysis of chromatin interactions. Nature 485, 376–380 10.1038/nature11082 22495300 PMC3356448

[B9] Doi M. Edwards S. F. (1984). The Theory of Polymer Dynamics. Oxford: Clarendon Press

[B10] Emanuel M. Radja N. Henriksson A. Schiessel H. (2009). The physics behind the larger scale organization of DNA in eukaryotes. Phys. Biol. 6:025008–025018 10.1088/1478-3975/6/2/025008 19571359

[B11] Fraser P. Bickmore W. (2007). Nuclear organization of the genome and the potential for gene regulation. Nature 447, 413–417 10.1038/nature05916 17522674

[B12] Gerton J. L. Hawley R. S. (2005). Homologous chromosome interactions in meiosis: diversity amidst conservation. Nat. Rev. Genet. 6, 477–487 10.1038/nrg1614 15931171

[B13] Kalhor R. Tjong H. Jayathilaka N. Alber F. Chen L. (2011). Genome architectures revealed by tethered chromosome conformation capture and population-based modeling. Nat. Biotechnol. 30, 90–98 10.1038/nbt.2057 22198700 PMC3782096

[B14] Lanctot C. Cheutin T. Cremer M. Cavalli G. Cremer T. (2007). Dynamic genome architecture in the nuclear space: regulation of gene expression in three dimensions. Nat. Rev. Genet. 8, 104–115 10.1038/nrg2041 17230197

[B15] Langowski J. (2006). Polymer chain models of DNA and chromatin. Eur. Phys. J. E Soft Matter 19, 241–249 10.1140/epje/i2005-10067-9 16547610

[B16] Lieberman-Aiden E. van Berkum N. L. Williams L. Imakaev M. Ragoczy T. Telling A. (2009). Comprehensive mapping of long-range interactions reveals folding principles of the human genome. Science 326, 289–293 10.1126/science.1181369 19815776 PMC2858594

[B17] Marenduzzo D. Micheletti C. Cook P. R. (2006). Entropy-driven genome organization. Biophys. J. 90, 3712–3721 10.1529/biophysj.105.077685 16500976 PMC1440752

[B18] Mateos-Langerak J. Bohn M. de Leeuw W. Giromus O. Manders E. M. M. Verschure (2009). Spatially confined folding of chromatin in the interphase nucleus. Proc. Natl. Acad. Sci. U.S.A. 106, 3812–3817 10.1073/pnas.0809501106 19234129 PMC2656162

[B19] Mirny L. A. (2011). The fractal globule as a model of chromatin architecture in the cell. Chromosome Res. 19, 37–51 10.1007/s10577-010-9177-0 21274616 PMC3040307

[B20] Misteli T. (2007). Beyond the sequence: cellular organization of genome function. Cell 128, 787–800 10.1016/j.cell.2007.01.028 17320514

[B21] Nicodemi M. Jensen H. J. (2001). Creep of superconducting vortices in the limit of vanishing temperature: a fingerprint of off-equilibrium dynamics. Phys. Rev. Lett. 86, 4378–4381 10.1103/PhysRevLett.86.4378 11328179

[B22] Nicodemi M. Prisco A. (2007). A symmetry breaking model for X chromosome inactivation. Phys. Rev. Lett. 98:108104 10.1103/PhysRevLett.98.108104 17358571

[B23] Nicodemi M. Prisco A. (2009). Thermodynamic pathways to genome spatial organization in the cell nucleus. Biophys. J. 96, 2168–2177 10.1016/j.bpj.2008.12.3919 19289043 PMC2717292

[B24] Nicodemi M. Panning B. Prisco A. (2008). A thermodynamic switch for chromosome colocalization. Genetics 179, 717–721 10.1534/genetics.107.083154 18493085 PMC2390650

[B25] Nora E. P. Lajoie B. R. Schulz E. G. Giorgetti L. Okamoto I. Servant N. (2012). Spatial partitioning of the regulatory landscape of the X-inactivation centre. Nature 485, 381–385 10.1038/nature11049 22495304 PMC3555144

[B26] Pombo A. Branco M. R. (2007). Functional organisation of the genome during interphase. Curr. Opin. Genet. Dev. 17, 451–455 10.1016/j.gde.2007.08.008 17920259

[B27] Scialdone A. Barbieri M. Pallotti D. Nicodemi M. (2011a). Mean-field theory of the symmetry breaking model for X chromosome inactivation. Prog. Theor. Phys. Suppl. 191:40 10.1143/PTPS.191.40

[B28] Scialdone A. Cataudella I. Barbieri M. Prisco A. Nicodemi M. (2011b). Conformation regulation of the X chromosome inactivation center: a model. PLoS Comp. Biol. 7:e1002229 10.1371/journal.pcbi.1002229 22046112 PMC3203058

[B29] Sexton T. Yaffe E. Kenigsberg E. Bantignies F. Leblanc B. Hoichman M. (2012). Three-dimensional folding and functional organization principles of the Drosophila genome. Cell 148, 458–472 10.1016/j.cell.2012.01.010 22265598

[B30] Shopland L. S. Lynch C. R. Peterson K. A. Thornton K. Kepper N. von Hase J. (2006). Folding and organization of a contiguous chromosome region according to the gene distribution pattern in primary genomic sequence. J. Cell Biol. 174, 27–38 10.1083/jcb.200603083 16818717 PMC2064156

[B31] Tark-Dame M. van Driel R. Heermann D. (2011). Chromatin folding–from biology to polymer models and back. J. Cell Sci. 124, 839–845 10.1242/jcs.077628 21378305

[B32] Tarzia M. Fierro A. Nicodemi N. Coniglio A. (2004). Glass transition in granular media. Europhys. Lett. 66, 531–537 10.1209/epl/i2004-10015-y

